# Contribution of NOTCH signaling pathway along with TNF-α in the intestinal inflammation of ulcerative colitis 

**Published:** 2019

**Authors:** Mahsa Ghorbaninejad, Raheleh Heydari, Parvaneh Mohammadi, Shabnam Shahrokh, Mehrdad Haghazali, Binazir Khanabadi, Anna Meyfour

**Affiliations:** 1 *Basic and Molecular Epidemiology of Gastrointestinal Disorders Research Center, Research Institute for Gastroenterology and Liver Diseases, Shahid Beheshti University of Medical Sciences, Tehran, Iran*; 2 *Gastroenterology and Liver Diseases Research Center, Research Institute for Gastroenterology and Liver Diseases, Shahid Beheshti University of Medical Sciences, Tehran, Iran *; 3 *Rajaie Cardiovascular Medical and Research Center, Iran University of Medical Sciences, Tehran, Iran*

**Keywords:** Ulcerative colitis, TNF-α, NOTCH signaling, HES1

## Abstract

**Aim:**

The aim of this study was to determine gene expression levels of TNF-α, NOTCH1, and HES1 in patients with UC.

**Background:**

Intestinal inflammation and epithelial injury are the leading actors of inflammatory bowel disease (IBD), causing an excessive expression of pro-inflammatory cytokines such as TNF-α. Also, target genes of NOTCH signaling are involved in the regulation of intestinal homeostasis. Previous studies have demonstrated that TNF-α increases in ulcerative colitis (UC) patients, but the relationship between TNF-α and NOTCH signaling pathway in UC etiopathology needs further study.

**Methods:**

Twelve active UC patients and twelve healthy controls were enrolled in this study. RNA was extracted and the mRNA expression levels of TNF-α, NOTCH1, and HES1 were examined using real-time PCR analyses. Further, transcriptome data deposited in Gene Expression Omnibus (GEO) database were analyzed to detect the differential expression of TNF superfamily and NOTCH1 gene in IBD patients. Finally, the interaction of TNF-α and NOTCH signaling was obtained from The SIGnaling Network Open Resource 2.0 (SIGNOR 2.0) database.

**Results:**

The transcription levels of TNF-α, NOTCH1, and HES1 genes were significantly elevated in UC patients compared with control (p < 0.05). In addition, GEO results confirmed our expression results. SIGNOR analysis showed that TNF-α interacts with NOTCH signaling components.

**Conclusion:**

Based on our data, we observed that NOTCH1 and HES1 in co-operation of TNF-α, may play an important role in pathogenesis of UC. The members of NOTCH signaling pathway can be ideal candidates to target the therapy of IBD.

## Introduction

 Inflammatory bowel disease (IBD) is an idiopathic chronic disorder whose incidence has been growing in the last decades around the world. IBD is mainly divided into two major forms: Crohn’s disease (CD) and ulcerative colitis (UC). IBD incidence is estimated to be approximately 1 in 1000 of the population (1). UC starts in the rectum and proceeds proximally in a uniform manner and is characterized by pathological signs such as mucosal and submucosal lesions. The prevalence rate of this disease is the highest in North America with 249 per 100,000 persons and 505 per 100,000 persons in Europe (2, 3). Immune response dysregulation and gut microbiome dysbiosis combined with genetic and environmental factors are involved in the pathogenesis of this recurrent intestinal inflammatory disorder. One of the most important factors implicated in etiopathogenesis of UC is mucosal insufficiency which results in abnormalities of mucosal epithelial cell proliferation, increase in intestinal epithelial permeability, tight junction weakness, aberrant mucin production, and dysregulated epithelial cell metabolism (4). Furthermore, it has been elucidated that immune cells produce pro-inflammatory cytokines which are essential in IBD pathogenesis. Tumor necrosis factor alpha (TNF-α) is one of the most important inflammatory cytokines that plays a pivotal role in the continuous immune dysregulation in the inflamed intestine (5). TNF-α release is regulated by inactive rhomboid protein 2 (iRhom2); in a study, both of them were upregulated in the colon of IBD patients (6). Furthermore, the UC path mechanism is linked to gene expression alterations in several signaling pathways not fully understood so far. NOTCH signaling pathway is a highly conserved system in many multicellular organisms (7). Indeed, NOTCH signaling is a master regulator of cell fate determination during intestinal homeostasis, indicating its pivotal role in pathogenesis of intestinal diseases. Notably, both aberrant expression and dysregulation of NOTCH signaling pathway genes are critically linked to the pathogenesis of several diseases (8-12). The expression of notch genes such as Notch1, Notch2, Hes1, and Jagged1 are enriched in crypts (13). In addition, overexpression of the Notch signaling pathway genes has been reported in hyperplastic crypts from inflamed intestines cells of DSS-colitis mice (7, 14). It has been shown that NOTCH signaling components are involved in inflammatory processes of some autoimmune diseases such as Rheumatoid arthritis (RA) and Systemic lupus erythematosus (SLE)(15). According to the studies, TNF-α through NFB pathway has crosstalk with Notch signaling. TNF-α induces the translocation of intracellular domain of Notch protein (NICD) into the nucleus of rheumatoid synovial fibroblasts, which in turn stimulates the expression of Notch-1, Notch-4, and Jagged-2 genes in rheumatoid arthritis tissues (16, 17). 

In another study, Zhang et al. observed that Notch signaling pathway plays an important role in activation of macrophage M2b polarization through enhancing NFB translocation into the nucleus in SLE disease. Inhibition of Notch signaling pathway resulted in ameliorated murine lupus (18). Nevertheless, the correlation of TNF-α with NOTCH1 target gene such as HES1 has not been reported in the context of Iranian patients with UC. In this study, we aimed to study the expression of NOTCH1 and HES1 as the main members of NOTCH signaling pathway as well as TNF- cytokine in patients suffering UC. Also, we analyzed high-throughput dataset to confirm the correlation of NOTCH signaling pathway and inflammation. Our findings may improve the etiopathogenesis of the IBD disease and highlight potential therapeutic targets for IBD treatment. 

## Methods

**Study Population and Sample Collection**

Samples were collected from UC and healthy subjects recruited from gastrointestinal and liver diseases clinic of Shahid Beheshti University of medical sciences, Tehran, Iran. The subjects with extra intestinal manifestation, other autoimmune diseases (ie. psoriasis, PSC, AIH, PBC, cirrhosis) were excluded from UC group. Subjects with significant findings at endoscopy (ie. ulcers, atypia) were excluded from the control groups. Written informed consent was obtained from patients prior to sample collection. Colonic mucosal biopsies were obtained during colonoscopy from 12 UC patients and 12 healthy controls. Disease activity was determined based on clinical and endoscopic findings. Control samples were obtained from normal colon tissue of healthy individuals undergoing colonoscopy (Table 1). Twelve patients were in the active-phase of disease. Biopsies were snap-frozen in liquid nitrogen prior to storage at -80˚C. 

**Table 1:** The clinic demographic characteristics of IBD patients

Variables	patients	Controls
Age (year)	40.58±14.57^*^	41.08±15.20
Sex Male Female	8 (66.6)^†^ 4 (33.4)	6 (50) 6 (50)
BMI (kg/m2)	27.49±5.27	25.94±4.53
Family history Positive Negative	1 (8.4) 11(91.6)	0 0
History of surgery Positive Negative	0 0	0 0
Smoking status Smoker Non-smoker	2 (16.7) 10 (83.3)	1(8.4) 11 (91.6)
Disease duration (month)	59±32.1	0

* mean ± standard deviation; ^†^ Number (%)

**RNA extraction and cDNA synthesis**

After tissue collection, RNA was extracted from snap-frozen biopsies using TRIzol (Invitrogen, USA) according to the manufacturer’s instructions. Any potential DNA contamination was removed by treating the extracted RNA with RNase-free DNase (EN0521- Thermo Scientific, Germany). cDNA synthesis was performed using RevertAid H Minus First Strand cDNA Synthesis Kit (K1632- Thermo Scientific, Germany) according to the manufacturer's instruction. The synthesized cDNA was stored a t -20°C for further experiments.

**Quantitative real-time polymerase chain reaction (qRT-PCR)**

mRNA expression levels of candidate genes were quantified by qRT-PCR as previously described (19). Briefly, q-PCR reactions were performed in duplicate on a Rotor Gene Q System (QIAGEN, Germany) using SYBR Green master mix (Ampliqon, Denmark). The calculation was performed using REST analysis software (QIAGEN, Germany). The relative expression of target genes was calculated by the comparative cycle threshold method (ΔΔCt). Gene Runner (version 3.05; www.generunner.net), and Perl Primer software (version v1.1.20; perlprimer.sourceforge.net) were used to design specific primers for *NOTCH1, HES1, TNF-α*, and *GAPDH.* These sequences were analyzed by Nucleotide Blast and Primer Blast in the NCBI database (http://blast.ncbi.nlm.nih.gov/). *GAPDH* was the housekeeping gene. The detailed information about the primers has been presented in Table 2. 

Table 2. List of primers used for quantitative real time (qRT)-PCR.

**Bioinformatics analysis**

The microarray expression data of GSE131359 and GSE64131 were downloaded from Gene Expression Omnibus (GEO) database (http://www.ncbi.nlm.nih.gov/geo) using keywords of “IBD disease” or “Ulcerative colitis”. The dataset GSE131359 included 3 IBD samples and 3 healthy controls, all coming from colon biopsies at the time of diagnosis. Another dataset, GSE64131, contained 4 UC samples and 2 healthy controls all obtained from human colon. Only the gene expression data of samples which had been diagnosed as UC by colonoscopy were used for further analysis. Then, the selected datasets were manually checked to select those studies that only included samples obtained from human colon tissue (but not liquid biopsy) for subsequent analysis (20, 21). In addition, the Signaling Network Open Resource (SIGNOR) database (available online at http://signor.uniroma2.it) was applied to analyze possible interactions between the members of NOTCH signaling pathway and TNF-α protein. 

**Table 2:** List of primers used for quantitative real time (qRT)-PCR

Gene	Primer sequence 5́ → 3ʹ	Product size
*NOTCH1*	F: CAGACCCACACCCAGTA R: GGCAACGTCAACACCTT	114
*HES1*	F: GGCTAAGGTGTTTGGAGG R: TGTTGCTGGTGTAGACGG	119
TNF-α	F: CCATGTTGTAGCAAACCCT R: GGACCTGGGAGTAGATGAG	145
*GAPDH*	F: CTCATTTCCTGGTATGACAACGA R: CTTCCTCTTGTGCTCTTGCT	121

**Figure 1:**
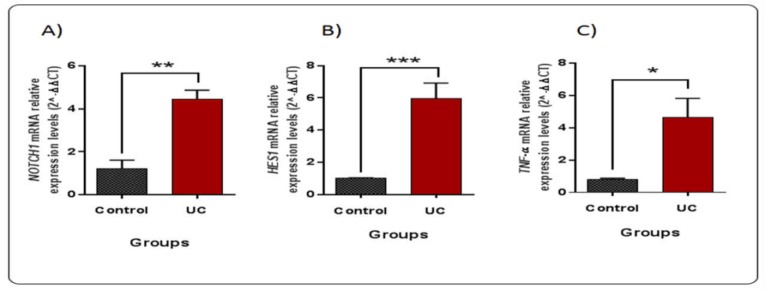
mRNA expression levels of NOTCH1 (A), HES1 (B), and TNF-α genes(C) in UC patients compared to healthy controls (*n* = 12); The results are expressed as 2^-ΔΔ^^𝐶^ (mean ± SEM). Means labeled with asterisks show significant differences versus control group in ∗p < 0.05, ∗∗p < 0.01, and ∗∗∗p < 0.001

**Figure 2:**
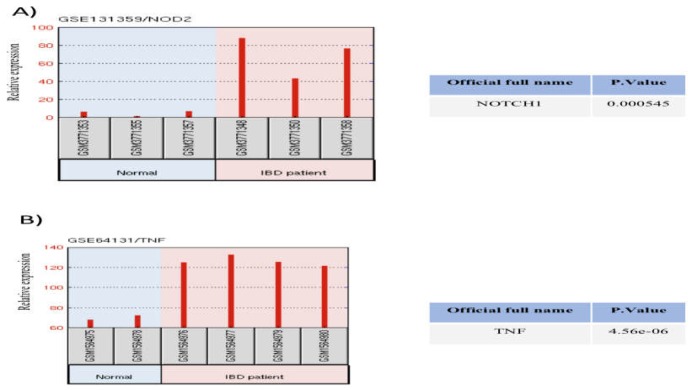
NOTCH1 and TNF mRNA expression in patients with UC and healthy controls; Data obtained from GEO datasets which are available at (http://www.ncbi.nlm.nih.gov/geo)

**Statistical analysis**

Statistical analyses were performed in Graphpad prism 6 software. The results were reported as mean ± SEM. Data were analyzed using a two-tailed unpaired student’s t-test. ∗p < 0.05, ∗∗p < 0.01, and ∗∗∗p < 0.001 indicated statistical significance. 

## Results

**Demographic information**

The 12 UC patients in the flare-up phase with the mean age of 40.58±14.57 years were included in the study including 8 (66.6%) males and 4 (33.4%) females. The mean value of body mass index (BMI) of the patients was 27.49±5.27 kg/m2. The mean disease duration was 59±32.1 months ranging from 12 to 120 months. The control group consisted of 12 healthy individuals, 6 (50%) males and 6 (50%) females with the mean age of 41.08±15.20 years. The comparison of the demographic characteristics between patients and control groups did not show any significant differences (age: p=0.93. BMI: p=0.46 and gender: p=0.29). The demographic characteristics of UC patients and healthy control groups have been reported in Table 1.

TNF-α and NOTCH signaling related genes showed the same expression pattern in ulcerative colitis

To quantify the relative expression level of TNF-α gene as the inflammatory marker gene, quantitative real-time PCR was performed for UC and healthy control groups. TNF-α was significantly up-regulated in UC patients (r=4.63, p=0.034) (Figure 1A). Next, the expression levels of NOTCH1and HES1 as two pivotal genes of NOTCH signaling pathway were analyzed in UC patients and healthy individuals. As illustrated in Figure 1B and C, the expression level of NOTCH1 and HES1, like TNF-α, increased in UC patients in comparison to the healthy control group (r=4.44, p=0.020), (r=5.94, p=0.0028).

**Figure 3:**
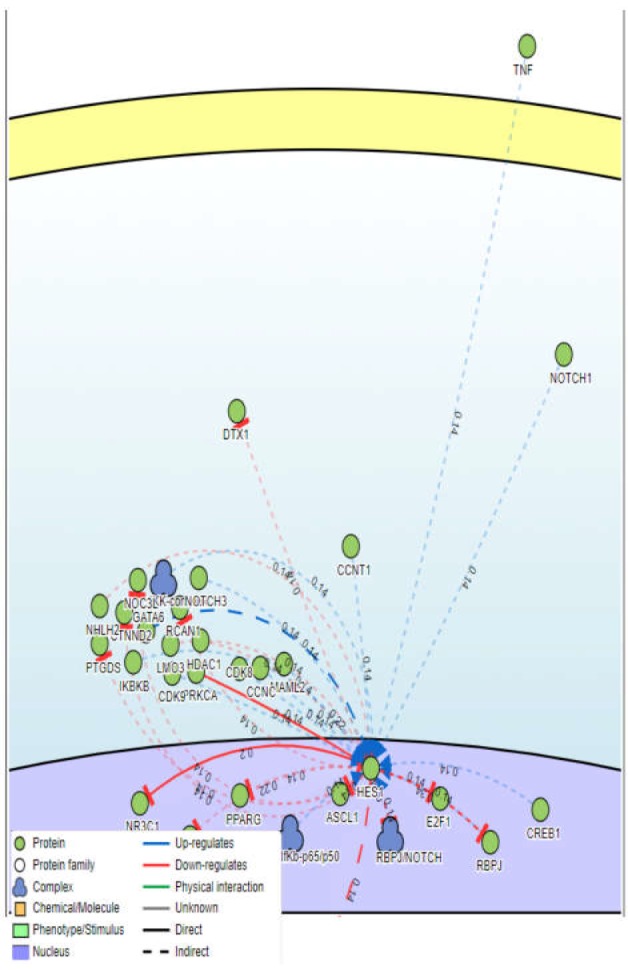
Curated interactions for HES1, NOTCH1, and TNF-α genes using SIGNOR database

**A data mining approach confirmed the relationship between inflammation and NOTCH signaling pathway.**

To confirm the experimental results, we analyzed NOTCH1 and TNF mRNA expression using the NCBI GEO database. Gene expression analysis of GSE131359 and GSE64131 datasets indicated that the inflamed tissues of UC patients had higher genes expression levels of NOTCH1 and TNF compared to healthy controls (Figure 2). Furthermore, we investigated possible interactions between the members of NOTCH signaling pathway and TNF-α protein using SIGNOR database. TNF-α as a pro-inflammatory cytokine led to increased HES1, a family of basic helix-loop-helix gene and a target gene of NOTCH signaling. On the other hand, several lines of evidence have proposed that NOTCH target gene such as HES1 can be activated by a constitutive active from NOTCH 1 (Figure 3). TNF-α and NOTCH 1 have a synergistic effect which promote the expression of HES1 gene. These results showed that NOTCH signaling interacts with inflammatory cytokine resulting in HES1 activation.

**Figure 4:**
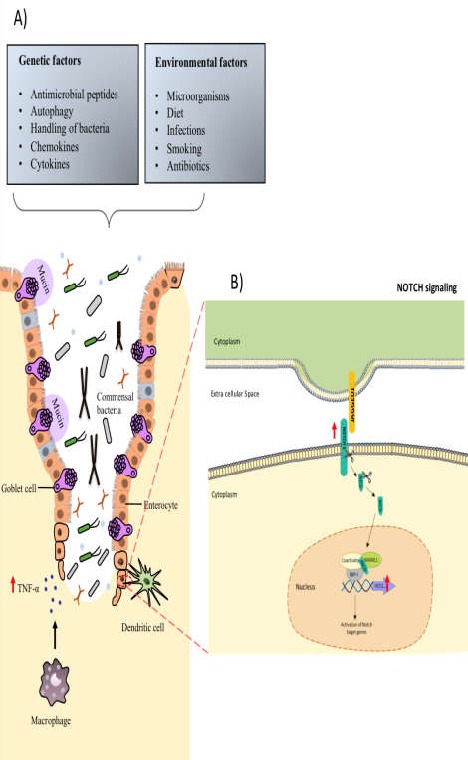
The crucial role of inflammatory cytokine, TNF-α, and NOTCH signaling pathway in inflammatory bowel disease (IBD); A) Regulation of epithelial cell death in the intestine by TNF-α; Increased TNF-α levels can trigger epithelial cell death in the intestine, which potentially leads to barrier defects and invasion of harmful pathogens. Together, this may trigger the intestinal inflammation. B) The NOTCH cascade consists of NOTCH and NOTCH ligands, as well as intracellular proteins transferring the NOTCH signal to nucleus of the cells

## Discussion

In recent years, continuous efforts have been made to gain a deeper understanding of the pathophysiology of IBD, which is defined as a relapsing disorder of the gastrointestinal tract. IBD is pathologically characterized by mucosal inflammation and epithelial damage not due to identifiable pathogens (22). Multiple factors are involved in IBD, including host immunity, environmental factors, and several genes (Figure 4A) that cause alterations in the state of intestinal homeostasis of these patients. Studies have shown that several important signaling pathways not only maintain this intestinal homeostasis but also prevent uncontrolled intestinal inflammation in healthy individuals. Furthermore, Several lines of research have focused on how such an uncontrolled, deleterious immune responses may arise and persist in IBD patients (23). These studies suggest that TNF-α, as a major inflammatory cytokine, plays a critical role in the IBD pathogenesis (Figure 4A). Regulation of TNF-α release is promoted through inactive rhomboid protein 2 (iRhom2) both of which are upregulated in the colon of IBD patients (6). In line with these results, we investigated the expression pattern of TNF-α in Iranian patients with UC. Our data showed a significant increase in TNF-α expression level in UC patients group compared to control samples. According to the fundamental role of TNF-α during the active phase of UC as well as lack of response to anti-TNF therapy in some UC patients (24), it seems that another dysregulated signaling pathway in parallel with TNF-α is involved in IBD pathogenesis. One of the most pivotal signaling pathways can be NOTCH signaling due to its fundamental role in maintaining the proliferation and differentiation of colonic epithelium (7). Notably, aberrant expression of the NOTCH signaling pathway in the UC leads to increased expression of the transcriptional factor, HES1, in the human colon cell line followed by suppression of intestine epithelial cells differentiation into goblet cells and weakening the mucus barrier (Figure 4B) (25). Furthermore, Kawamoto et al. observed that NOTCH signaling and TNF-α-induced NFκB signaling had a synergistic effect on human intestinal epithelial cell lines and promoted the expression of specific genes such as ubiquitin D (UBD). UBD expression was dependent on NOTCH and TNF-α, and was also upregulated in IBD patients (26). Considering the possible relationship between NOTCH1 and HES1 function and UC disease, we measured the expression level of NOTCH1 and HES1 as two critical components of NOTCH signaling pathway in the colon biopsies of active UC patients and healthy controls. Our results demonstrated that the expression of NOTCH1 and HES1 mRNA similar to TNF-α were up-regulated in Iranian patients with UC. Supporting these results, we examined the expressional changes of NOTCH1 and TNF mRNAs between IBD patients and healthy individuals using GEO databases. Bioinformatics analysis of different datasets revealed that TNF and NOTCH1 significantly increased in IBD patients. In addition, our protein-protein interaction analysis based on SIGNOR database offered a valuable network containing experimentally validated relationships between NOTCH signaling components, NOTCH1, as well as HES1 and TNF proinflammatory cytokine. According to bioinformatics analysis, up-regulation of TNF-α along with NOTCH1 leads to overexpression of HES1 in the active form of UC. In other words, NOTCH1 in co-operation with TNF-α may have an important role in intestinal inflammation. 

We can conclude that NOTCH signaling can be a clinical drug target for IBD treatment. NOTCH signaling inhibitors in combination with anti-TNF antibodies can be examined in both in vitro/in vivo IBD models. Definitely, a deeper understanding of this context is essential in the design of cell type-specific drugs targeting the NOTCH pathway.
